# The Chivalrous Lord Chancellor

**Published:** 1920-06-26

**Authors:** 


### The Chivalrous Lord Chancellor.

Judging by the debate in the House of Lords on the London Juvenile Courts Bill, there seems to have been a good deal of unnecessary pother about the proposals. The Lord Chancellor's defence of the measure broadly summarised amounts to this: that because hitherto the London stipendiary magistrates have dealt with children's cases, women magistrates of London are at present deprived of the privilege of exercising their newly-gotten magisterial functions. The stipendiaries have done their work well, but women magistrates in the provinces have a say in the matter, so why not women magistrates in London? And, moreover, has not a good deal of labour been expended over the measure? Therefore, why make it all for naught by refusing to pass the Bill? This is all very chivalrous to the women and to the departmental authors of Bills, but it is not a sex question at all—for lay women magistrates in London are in exactly the same position as men—and it is a new doctrine that Bills should not be rejected because many excellent officials have worked so hard upon them. At the same time, there is much to be said for a provision which makes use of women's particular experiences and knowledge for dealing with young offenders. The main objection to the Bill is the apparent intention to set up a central London court for children to which the offenders would be brought. It was against this that most of the opposition was directed, but the Lord Chancellor dispelled this idea by declaring that, in addition to the central court, many branch courts would also be set up where experience and convenience suggested, and the present system would not be in the least altered in this respect. This explanation succeeded in getting a second reading of the Bill, but it might just as well have been given while the controversy was taking place through the Press—a controversy in which responsible authorities participated.

